# Towards a better understanding of the role of nectar-inhabiting yeasts in plant–animal interactions

**DOI:** 10.1186/s40694-019-0091-8

**Published:** 2020-01-08

**Authors:** Joon Klaps, Bart Lievens, Sergio Álvarez-Pérez

**Affiliations:** Department of Microbial and Molecular Systems (M2S), Laboratory for Process Microbial Ecology and Bioinspirational Management (PME & BIM), KU Leuven, Willem De Croylaan 46, Leuven, 3001 Belgium

**Keywords:** Floral mycobiome, *Metschnikowia*, Nectar, Plant–insect interactions, Pollinators, Volatile organic compounds

## Abstract

Flowers offer a wide variety of substrates suitable for fungal growth. However, the mycological study of flowers has only recently begun to be systematically addressed from an ecological point of view. Most research on the topic carried out during the last decade has focused on studying the prevalence and diversity of flower-inhabiting yeasts, describing new species retrieved from floral parts and animal pollinators, and the use of select nectar yeasts as model systems to test ecological hypotheses. In this primer article, we summarize the current state of the art in floral nectar mycology and provide an overview of some research areas that, in our view, still require further attention, such as the influence of fungal volatile organic compounds on the foraging behavior of pollinators and other floral visitors, the analysis of the direct and indirect effects of nectar-inhabiting fungi on the fitness of plants and animals, and the nature and consequences of fungal-bacterial interactions taking place within flowers.

## What is the link between flowers and fungi?

Flowers are essential structures in the reproductive cycle of angiosperms. Accordingly, most animal-pollinated plants spend abundant resources to make their flowers attractive to pollinators by, for example, displaying alluring colors, secreting specific odors, forming characteristic shapes, and providing nutritional and non-nutritional rewards [1–4].

When animals visit flowers (e.g. to feed on nectar, seek shelter or use these as mating/nesting places), pollen can get attached to their body surfaces and subsequently be spread to new flowers [1, 4]. Pollinators and non-pollinating visitors are not sterile but carry diverse microbial communities, particularly consisting of bacteria and fungi, that may disperse to nectar and the surface of other floral parts such as the corolla, stamens, and pistil when visiting flowers [5–11]. The dispersal of microbes from flower to flower by animal vectors is a dynamic process that keeps ongoing during the flower lifetime [6, 9, 10]. Nevertheless, observations of microbial community assembly in floral nectar suggest that dispersed microorganisms interact by competing for niche space and that the first microbial species to colonize the nectar precludes the successful establishment of subsequent immigrants [12, 13].

Traditionally, the mycological study of flowers has mostly focused on flower-infecting fungi, which use the nectaries and other flower structures to penetrate into plant tissues and eventually invade other organs. Remarkably, in some cases fungal infection leads to the formation of pseudo flowers (flower mimics) that can attract pollinators, via visual and/or olfactory cues, to get their infectious propagules spread to new plants [14]. A detailed review of plant-parasitic fungi is nevertheless beyond the scope of this primer article, but has been extensively covered elsewhere [e.g. 14–16]. Instead, we will focus on recent advances and future prospects in the ecological study of nectar yeasts and, to a minor extent, other flower-inhabiting, non-pathogenic fungi.

## Why is it worth studying flower-inhabiting fungi?

Nearly 90% of all plant species, including 75% of domesticated crops, profit from animal-mediated pollination [17]. As the main reward for their services, flowers offer nectar to the visiting pollinators providing them with sugars and other nutrients [17, 18]. Given the nutrient-rich nature of nectar and other floral rewards, microorganisms are commonly found in the flowers of a wide diversity of plant species worldwide [7, 8, 19–29]. Flower-inhabiting microbes can alter the chemical composition of their habitat by consuming the available nutrients and/or releasing metabolic by-products [30–32], which, in turn, may affect pollinators’ foraging behavior and have an impact on the reproductive success of the plant (see below). Furthermore, the disease-suppressing capabilities of some flower-inhabiting fungi such as *Metschnikowia pulcherrima*, *Cryptococcus* spp., and *Aureobasidium pullulans* (e.g. by antagonizing phytopathogenic microorganisms), should not be overlooked [33, 34]. In this way, the downstream effect of microbes in floral biology may have important consequences on plant-animal interactions, plant fitness and plant health [19, 28, 35], and eventually have a relevant impact on agriculture, ecosystem dynamics and plant conservation. Finally, flower-inhabiting fungi have a huge potential in industrial applications as demonstrated, for example, for second-generation bioethanol production [36]. Therefore, it is not surprising that flower microbiology is currently receiving a greater interest. An overview of flower-inhabiting microbes and their effects on plants and the animal visitors of flowers is presented in the Additional file 1.

## Three advances in the last decade

### Prevalence and diversity of flower-inhabiting yeasts

Mycological study of flowers and their pollinators dates back more than a century. For example, in 1884, Boutroux [37] investigated the presence of yeasts (*‘ferments alcooliques’*) in flowers, fruits and insects, and assessed their species diversity by detailed morphological and physiological characterization. Since then, investigations carried out by different research groups, mostly during the last decade, have revealed that the flowers of phylogenetically-diverse plant species around the world are a habitat for fungi and, in particular, yeasts from the genus *Metschnikowia* (Ascomycota) [19, 20, 24, 27–29, 38–42]. Other yeast genera that are commonly found in nectar and floral surfaces include *Aureobasidium*, *Candida*, *Clavispora*, *Cryptococcus*, *Debaryomyces*, *Hanseniaspora*, *Kodamaea*, *Papiliotrema*, *Rhodotorula*, *Starmerella*, *Sporobolomyces*, and *Wickerhamiella*, but most of these other yeasts are generally less abundant than *Metschnikowia* spp. [19, 20, 24, 27–29, 38, 40–43]. Besides, it is foreseeable that this list of genera will keep expanding as new studies of fungal presence in flowers are increasingly published.

So far, most mycological surveys of flowers have focused on the yeast communities associated to floral nectar, whereas the presence of fungi in other floral parts has only been addressed in a few instances. For example, Pusey et al. [34] characterized the epiphytic populations of yeasts and yeast-like fungi on apple (*Malus pumila* cultivars ‘Gala’ and ‘Red Delicious’) stigmas, and hypanthia during primary bloom and identified some fungi, including *Cryptococcus* spp., that were able to suppress the bacterial species *Erwinia amylovora* (causal agent of fire blight in pome fruit trees). Furthermore, Pozo et al. [44] analyzed the occurrence of yeasts in the outer and inner corolla, pollen, and nectar of *Digitalis obscura* and *Atropa baetica* plants from south-eastern Spain, and found for both host species a higher yeast species richness in corolla samples than in pollen and nectar. More recently, Klaps [29] studied the diversity of culturable yeasts inhabiting the nectar, stamina and styles of *Metrosideros polymorpha*, a tree endemic to Hawaii (USA). The results of this latter study showed that *M. polymorpha* flowers are inhabited by species-poor yeast communities that are dominated by ascomycetous taxa. Additionally, the yeast communities associated to specific floral structures of *M. polymorpha* showed differences in species richness and phylogenetic diversity, both of which were higher for stamina and styles than for nectar [29]. Such microhabitat-dependent variation in species richness may be due not only to the large morphological and physiological differences occurring within flowers, but also to the filtering effect of microbial diversity exerted by specific floral microhabitats such as floral nectar [19, 29, 45–47].

Very limited attention has been paid so far to the fungal endophytes of flowers, with most published studies focusing on the presence of specific fungal pathogens or mycotoxin producers in the flowers of economically-important plants, such as eucalyptus trees (*Eucalyptus globulus*, [48]) and pasture grasses (*Festuca* spp. and *Lolium* spp., [49]). Additionally, Martinson et al. [50] examined the diversity and composition of the endophytic fungal communities associated with fig (*Ficus* spp.) flowers at different developmental stages. Non-significant differences were found in this latter study in the fungal communities associated with non-pollinated flowers of six different species of *Ficus*, or between gall- and seed-flowers (which are likely to receive wasp’s eggs and pollen, respectively). However, the endophytic communities differed significantly in fig flowers after pollination vs. before pollination, and between *Ficus* lineages with active vs. passive pollination syndromes [50].

### Flowers as reservoirs of undescribed fungal taxa

Flowers and their animal visitors are being increasingly recognized as a rich source of undescribed fungal species [19, 29, 43]. For example, the study of these habitats has led to the discovery of more than 50 new yeast species during the last decade, most of which were classified within the ascomycetous genera *Metschnikowia*, *Wickerhamiella*, *Starmerella* and *Kodamaea* [43, 51–60]. In contrast, descriptions of new species of mycelial fungi obtained from flowers are much scarcer (but see, for example, [61]).

An important limitation of some descriptions of new species of flower-inhabiting fungi is that isolates were obtained by enrichment culture or maceration of whole flowers or fragments of them (e.g. [58, 60, 62]), without further information about the specific microhabitats hosting the new species. Furthermore, evaluating the biogeographic distribution of floral-inhabiting fungi remains challenging because of the limited number of studies performed so far in some locations and, in particular, in tropical regions where most angiosperm’s diversity is distributed [29, 43]. In this regard, de Vega et al. [43] predicted that nectar yeast diversity should increase in habitats with a higher phylogenetic diversity of plants and a concomitant higher diversity of functional pollinator guilds.

### Nectar yeasts as model systems in ecology

Nectar yeasts are currently considered a powerful study system for testing ecological theory of processes affecting community assembly, such as environmental filtering, dispersal, historical contingency, and meta community dynamics [6, 10, 13, 46, 63–65].

There are several characteristics that make nectar yeasts well suited for microcosm studies in ecology, including their short generation times, the relative simplicity of their communities (1.2 culturable yeast species/nectar sample on average [28]), and the fact that nectar habitats are arranged in a well-defined hierarchical structure of increasing complexity (nectaries within flowers, flowers within individual plants, plants within populations, and so on), thus allowing multiscale approaches [63]. Moreover, nectar yeast communities can be easily manipulated and monitored over time [63]. The potential of other flower-associated microbes (e.g. epiphytic communities of petals and other floral surfaces) as model systems in ecological research remains to be explored in detail, but the results obtained by Russell et al. [9] when analyzing how the foraging behavior of the bumble bee *Bombus impatiens* shapes the dispersal of the bacterium *Pseudomonas fluorescens* among and within natural and artificial flowers are promising in that regard. In particular, the authors observed that bee foraging behavior affected the acquisition and deposition of *P. fluorescens*, and that the microbes acquired from the corolla were mainly deposited on the corolla of other flowers, followed by the stamens, and least on the nectary/pistil [9].

## Three areas ripe for development

### Effect of fungal volatile organic compounds on animal behavior

The importance of flower-associated fungi for plant-animal mutualisms has only recently been explicitly addressed. Research on this issue is still limited in scope and mostly involves a few species of yeasts. Nevertheless, there is already enough evidence to conclude that flower-inhabiting yeasts can produce species-specific blends of volatile organic compounds (VOCs) that alter the behavior of pollinators and other floral visitors [66–70]. Production of VOCs attracting insects and other animals may be especially advantageous for specialist yeast species that strongly rely on animal vectors to travel to new habitats. For example, the VOCs emitted by the nectar specialists *M. reukaufii* and *M. gruessii* are attractive to the nectar-feeding aphid parasitoid *Aphidius ervi* (Hymenoptera), whereas those produced by yeast generalist species such as *Hanseniaspora uvarum* and *Sporobolomyces roseus* have a neutral or deterrent effect on the parasitoid [69, 70]. Similarly, results of controlled laboratory assays and field experiments have shown that *M. reukaufii* is either attractive [66, 71–73] or not deterrent to bee pollinators [74]. Rering et al. [66] found that antennal responses of honey bees were much greater in response to compounds like 2-butanol, which was only produced by *M. reukaufii*, than to the other compounds emitted by any of the tested species. Further research is needed to elucidate the actual effects of this compound. Additionally, it still remains difficult to predict the effects of yeast VOCs on floral visitors as they not only depend on the emitting and receiving species (i.e. yeast and animal, respectively), but also on the concentration of the compounds and their interaction with other compounds present in the VOC blend [75].

Notably, it has been recently observed that the chemical cues produced by epiphytic microbes (both yeasts and bacteria) occurring on the petals of flowers can mediate both learned and innate components of *Bombus impatients* preference, and that the learning of such microbial community cues is associative [67]. Likewise, *A. ervi* parasitoids can rapidly learn to associate the volatiles released by nectar yeasts with the presence of a suitable food source [70]. Additionally, it seems that *B. impatients* can respond differentially to olfactory vs. gustatory cues produced by nectar microbes [68]. In particular, the VOC blend produced by the acetic acid bacterium *Asaia astilbes* was found to be significantly more attractive to *B. impatients* than the mixture of VOCs produced by *M. reukaufii* but, nevertheless, the insect preferentially consumed the nectar fermented by the latter species [68]. Therefore, it seems that both associative learning and olfactory vs. gustatory cues may be involved in plant-animal signaling, but the specific action of individual compounds emitted by the different species of flower-inhabiting fungi remains to be further explored.

### Effect of flower-inhabiting fungi on plant and animal fitness

The limited research carried out so far on the effects of flower-inhabiting fungi on plant fitness has focused again on *M. reukaufii*. For example, Eisikowitch et al. [76] demonstrated that this yeast species inhibits pollen germination in *Asclepias syriaca* by causing the immediate death of the growing microgametophyte. Moreover, Herrera et al. [71] reported that experimental inoculation of *M. reukauffi* into the floral nectar of *Helleborus foetidus* resulted in a reduction of the number of pollen tubes in the style, fruit set, seed set, and mass of individual seeds produced, therefore having detrimental effects on pollination success and plant maternal fecundity. Such findings were interpreted as the combined consequence of a possible limitation of *H. foetidus* maternal fecundity in the study season due to pollen quality, and longer visits by pollinators to yeast-containing flowers that would increase the proportion of self-pollen in stigmatic pollen loads [71]. In contrast, Vannette et al. [77] found no detrimental effects of *M. reukauffi* on estimates of female fitness in the hummingbird-pollinated plant *Mimulus (Diplacus) aurantiacus*. Finally, Schaeffer and Irwin [72] did not find any evidence that inoculation of *Delphinium nuttallianum* flowers with *M. reukauffi* directly or indirectly affected female reproduction but, in contrast, the authors detected positive effects of yeast presence on pollen donation (i.e. male plant reproduction). All in all, it seems that the effect of nectar-inhabiting yeasts on plant fitness may depend not only on their direct effects on pollinators (see in previous subsection), but also on plant specific attributes such as flower morphology, plant mating system, the component of reproduction measured, and the pollen limitation experienced [28, 71, 72]. Additionally, the specific effect(s) on plant fitness of nectar inhabitants other than *M. reukauffi* and the fungal communities associated to other floral parts should be addressed in the future.

Knowledge about the effects of flower-inhabiting fungi on the fitness of animals is also very scarce. Nevertheless, Sobhy et al. [69] reported that the modification of nectar’s chemistry caused by *M. gruessii* and *M. reukaufii* had no apparent adverse effect on the longevity and survival of adult *A. ervi* individuals, whereas the parasitoids that fed on nectars fermented with *Aureobasidium pullulans*, *H. uvarum* or *S. roseus* showed shorter longevity and lower survival. A similar species-dependent effect of microbial modification of nectar on insect longevity has also been reported for nectar-inhabiting bacteria [78]. In any case, it remains to be established whether nectar microbes can also affect other life history parameters such as fecundity and oviposition frequency, or if other flower-visiting animals respond differently [69, 78].

### Fungal-bacterial interactions

Most studies on the flower–insect–microbe system to date have focused on yeasts, and it is only recently that also bacteria have been studied in this regard [9, 10, 13, 66–68, 74, 77–80]. Nevertheless, very limited attention has been given to potential fungal–bacterial interactions, even when recent evidence suggests that such interactions drive the assembly of nectar microbial communities and might affect plant–animal interplays [45]. The limited information currently available on the potential interactions taking place between flower-inhabiting fungi and bacteria mostly came from the analysis of co-occurrence patterns of nectar yeasts and bacteria [20], and the study of microcosms mimicking floral nectar [81]. Potential mechanisms of fungal–bacterium interactions in floral microhabitats worthy of being studied include the formation of physical complexes (e.g. cell aggregates, multi-species biofilms, and endosymbiotic associations), nutritional interactions (competition, syntrophy, cross-feeding, etc.), antibiosis, signaling-based interactions (e.g. quorum sensing), and horizontal gene transfer between fungal and bacterial cells [45].

## Conclusions

Despite recent advances, the study of the diversity and ecological significance of flower-inhabiting fungi is still in its infancy. So far, most research has focused on nectar yeasts, overlooking that pollinators generally encounter other floral structures while searching for the nectaries and, in some cases, they may actually seek rewards other than nectar, including pollen, oils, stigmatic secretions, and several non-nutritive rewards [1, 4, 9]. Despite recent advances in the field, mostly related to the diversity and taxonomic study of floricolous fungi, there is still limited information on the impact of fungal activity on plant reproduction and the behavioral responses of floral visitors. Given the huge ecological and economic importance of plant pollination at a global scale, we predict that the study of flower-inhabiting microbes will be a research priority in the near future.

## Supplementary information

**Additional file 1.** Poster providing an overview of flower-inhabiting fungi and their effects on the host plant and flower visitors.

## Data Availability

Not applicable.

## Abbreviation

VOCs: volatile organic compounds

## References

[1] Kevan PG, Capinera JL (2008). Pollination and flower visitation. Encyclopedia of entomology.

[2] Moyroud E, Glover BJ (2017). The physics of pollinator attraction. New Phytol.

[3] Schiestl FP (2015). Ecology and evolution of floral volatile-mediated information transfer in plants. New Phytol.

[4] Simpson BB, Neff JL (1981). Floral rewards: alternatives to pollen and nectar. Ann Missouri Bot Gard..

[5] Aizenberg-Gershtein Y, Izhaki I, Halpern M (2013). Do honeybees shape the bacterial community composition in floral nectar?. PLoS ONE.

[6] Hausmann SL, Tietjen B, Rillig MC (2017). Solving the puzzle of yeast survival in ephemeral nectar systems: exponential growth is not enough. FEMS Microbiol Ecol..

[7] Junker RR, Keller A (2015). Microhabitat heterogeneity across leaves and flower organs promotes bacterial diversity. FEMS Microbiol Ecol..

[8] Junker RR, Loewel C, Gross R, Dötterl S, Keller A, Blüthgen N (2011). Composition of epiphytic bacterial communities differs on petals and leaves. Plant Biol (Stuttg).

[9] Russell AL, Rebolleda-Gomez M, Shaible TM, Ashman T-L (2019). Movers and shakers: bumble bee foraging behavior shapes the dispersal of microbes among and within flowers. Ecosphere..

[10] Vannette RL, Fukami T (2017). Dispersal enhances beta diversity in nectar microbes. Ecol Lett.

[11] Zemenick AT, Rosenheim JA, Vannette RL (2018). Legitimate visitors and nectar robbers of *Aquilegia formosa* have different effects on nectar bacterial communities. Ecosphere..

[12] Peay KG, Belisle M, Fukami T (2012). Phylogenetic relatedness predicts priority effects in nectar yeast communities. Proc Biol Sci..

[13] Toju H, Vannette RL, Gauthier M-PL, Dhami MK, Fukami T (2018). Priority effects can persist across floral generations in nectar microbial metacommunities. Oikos..

[14] Ngugi HK, Scherm H (2006). Mimicry in plant-parasitic fungi. FEMS Microbiol Lett.

[15] Antonovics J (2005). Plant venereal diseases: insights from a messy metaphor. New Phytol.

[16] Ngugi HK, Scherm H (2006). Biology of flower-infecting fungi. Annu Rev Phytopathol.

[17] Roy R, Schmitt AJ, Thomas JB, Carter CJ (2017). Review: nectar biology: from molecules to ecosystems. Plant Sci.

[18] Nicolson SW, Thornburg RW, Nicolson SW, Nepi M, Pacini E (2007). Nectar chemistry. Nectaries and nectar.

[19] Aleklett K, Hart M, Shade A (2014). The microbial ecology of flowers: an emerging frontier in phyllosphere research. Botany..

[20] Álvarez-Pérez S, Herrera CM (2013). Composition, richness and nonrandom assembly of culturable bacterial-microfungal communities in floral nectar of Mediterranean plants. FEMS Microbiol Ecol.

[21] Álvarez-Pérez S, Herrera CM, de Vega C (2012). Zooming-in on floral nectar: a first exploration of nectar-associated bacteria in wild plant communities. FEMS Microbiol Ecol.

[22] Ambika Manirajan B, Ratering S, Rusch V, Schwiertz A, Geissler-Plaum R, Cardinale M, Schnell S (2016). Bacterial microbiota associated with flower pollen is influenced by pollination type, and shows a high degree of diversity and species-specificity. Environ Microbiol.

[23] Ambika Manirajan B, Maisinger C, Ratering S, Rusch V, Schwiertz A, Cardinale M, Schnell S (2018). Diversity, specificity, co-occurrence and hub taxa of the bacterial-fungal pollen microbiome. FEMS Microbiol Ecol..

[24] Belisle M, Peay KG, Fukami T (2012). Flowers as islands: spatial distribution of nectar-inhabiting microfungi among plants of *Mimulus aurantiacus*, a hummingbird-pollinated shrub. Microb Ecol.

[25] Fridman S, Izhaki I, Gerchman Y, Halpern M (2012). Bacterial communities in floral nectar. Environ Microbiol Rep..

[26] Herrera CM, de Vega C, Canto A, Pozo MI (2009). Yeasts in floral nectar: a quantitative survey. Ann Bot.

[27] Pozo MI, Herrera CM, Bazaga P (2011). Species richness of yeast communities in floral nectar of southern Spanish plants. Microb Ecol.

[28] Pozo M, Lievens B, Jacquemyn H, Peck RL (2015). Impact of microorganisms on the nectar chemistry, pollinator attraction and plant fitness. Nectar: production, chemical composition and benefits to animals and plants.

[29] Klaps J (2019). Analysis of yeast diversity in floral microhabitats from *Metrosideros polymorpha Gaud*.

[30] Herrera CM, García IM, Pérez R (2008). Invisible floral larcenies: microbial communities degrade floral nectar of bumblebee-pollinated plants. Ecology.

[31] Parachnowitsch AL, Manson JS, Sletvold N (2019). Evolutionary ecology of nectar. Ann Bot.

[32] Vannette RL, Fukami T (2018). Contrasting effects of yeasts and bacteria on floral nectar traits. Ann Bot.

[33] Duffy B, Vogelsanger J, Schoch B, Holliger E (2006). Biocontrol of *Erwinia amylovora* using a commercial yeast strain mixture. Acta Hortic.

[34] Pusey PL, Stockwell VO, Mazzola M (2009). Epiphytic bacteria and yeasts on apple blossoms and their potential as antagonists of *Erwinia amylovora*. Phytopathology..

[35] Rebolleda-Gómez M, Forrester NJ, Russell AL, Wei N, Fetters AM, Stephens JD, Ashman T-L (2019). Gazing into the anthosphere: considering how microbes influence floral evolution. New Phytol.

[36] Mukherjee V, Radecka D, Aerts G, Verstrepen KJ, Lievens B, Thevelein JM (2017). Phenotypic landscape of non-conventional yeast species for different stress tolerance traits desirable in bioethanol fermentation. Biotechnol Biofuels.

[37] Boutroux L (1884). Conservation des ferments alcooliques dans la nature. Annales des Sci Naturelles, Série IV, Botanique..

[38] Bartlewicz J, Lievens B, Honnay O, Jacquemyn H (2016). Microbial diversity in the floral nectar of *Linaria vulgaris* along an urbanization gradient. BMC Ecol.

[39] Brysch-Herzberg M (2004). Ecology of yeasts in plant-bumblebee mutualism in Central Europe. FEMS Microbiol Ecol.

[40] Canto A, Herrera CM, Rodriguez R (2017). Nectar-living yeasts of a tropical host plant community: diversity and effects on community-wide floral nectar traits. Peer J..

[41] Jacquemyn H, Lenaerts M, Brys R, Willems K, Honnay O, Lievens B (2013). Among-population variation in microbial community structure in the floral nectar of the bee-pollinated forest herb *Pulmonaria officinalis* L. PLoS ONE.

[42] Mittelbach M, Yurkov AM, Nocentini D, Nepi M, Weigend M, Begerow D (2015). Nectar sugars and bird visitation define a floral niche for basidiomycetous yeast on the Canary Islands. BMC Ecol.

[43] de Vega C, Albaladejo RG, Guzmán B, Steenhuisen SL, Johnson SD, Herrera CM, Lachance MA (2017). Flowers as a reservoir of yeast diversity: description of *Wickerhamiella nectarea* f.a. sp. nov., and *Wickerhamiella natalensis* f.a. sp. nov. from South African flowers and pollinators, and transfer of related *Candida* species to the genus *Wickerhamiella* as new combinations. FEMS Yeast Res..

[44] Pozo MI, Lachance MA, Herrera CM (2012). Nectar yeasts of two southern Spanish plants: the roles of immigration and physiological traits in community assembly. FEMS Microbiol Ecol.

[45] Álvarez-Pérez S, Lievens B, Fukami T (2019). Yeast–bacterium interactions: the next frontier in nectar research. Trends Plant Sci.

[46] Herrera CM, Canto A, Pozo MI, Bazaga P (2010). Inhospitable sweetness: nectar filtering of pollinator-borne inocula leads to impoverished, phylogenetically clustered yeast communities. Proc Biol Sci..

[47] Lievens B, Hallsworth JE, Pozo MI, Belgacem ZB, Stevenson A, Willems KA, Jacquemyn H (2015). Microbiology of sugar-rich environments: diversity, ecology and system constraints. Environ Microbiol.

[48] Lupo S, Tiscornia S, Bettucci L (2001). Endophytic fungi from flowers, capsules and seeds of *Eucalyptus globulus*. Rev Iberoam Micol..

[49] Sugawara K, Ohkubo H, Mikoshiba Y, Yamashita M (2004). Flowers for *Neotyphodium* endophytes detection: a new observation method using flowers of host grasses. Mycoscience..

[50] Martinson EO, Herre EA, Machado CA, Arnold AE (2012). Culture-free survey reveals diverse and distinctive fungal communities associated with developing figs (*Ficus* spp.) in Panama. Microb Ecol..

[51] Alimadadi N, Soudi MR, Wang SA, Wang QM, Talebpour Z, Bai FY (2016). *Starmerella orientalis* f.a., sp. nov., an ascomycetous yeast species isolated from flowers. Int J Syst Evol Microbiol..

[52] Amoikon TLS, Grondin C, Djéni TN, Jacques N, Casaregola S (2018). *Starmerella reginensis* f.a., sp. nov. and *Starmerella kourouensis* fa, sp. nov, isolated from flowers in French Guiana. Int J Syst Evol Microbiol..

[53] Daniel HM, Rosa CA, São Thiago-Calaça PS, Antonini Y, Bastos EM, Evrard P, Huret S, Fidalgo-Jiménez A, Lachance MA (2013). *Starmerella neotropicalis* f. a., sp. nov., a yeast species found in bees and pollen. Int J Syst Evol Microbiol..

[54] de Vega C, Guzmán B, Lachance MA, Steenhuisen SL, Johnson SD, Herrera CM (2012). *Metschnikowia proteae* sp. nov., a nectarivorous insect-associated yeast species from Africa. Int J Syst Evol Microbiol..

[55] de Vega C, Guzmán B, Steenhuisen SL, Johnson SD, Herrera CM, Lachance MA (2014). *Metschnikowia drakensbergensis* sp. nov. and *Metschnikowia caudata* sp. nov., endemic yeasts associated with Protea flowers in South Africa. Int J Syst Evol Microbiol..

[56] de Vega C, Albaladejo RG, Lachance MA (2018). *Metschnikowia maroccana* f.a., sp. nov., a new yeast species associated with floral nectar from Morocco. Int J Syst Evol Microbiol..

[57] Lachance MA, Vale HMM, Sperandio EM, Carvalho AOS, Santos ARO, Grondin C, Jacques N, Casaregola S, Rosa CA (2018). *Wickerhamiella dianesei* f.a., sp. nov. and *Wickerhamiella kurtzmanii* f.a., sp. nov., two yeast species isolated from plants and insects. Int J Syst Evol Microbiol..

[58] Li SL, Li ZY, Yang LY, Zhou XL, Dong MH, Zhou P, Lai YH, Duan CQ (2013). *Starmerella jinningensis* sp. nov., a yeast species isolated from flowers of *Erianthus rufipilus*. Int J Syst Evol Microbiol..

[59] Santos ARO, Leon MP, Barros KO, Freitas LFD, Hughes AFS, Morais PB, Lachance MA, Rosa CA (2018). *Starmerella camargoi* f.a., sp. nov., *Starmerella ilheusensis* f.a., sp. nov., *Starmerella litoralis* f.a., sp. nov., *Starmerella opuntiae* f.a., sp. nov., *Starmerella roubikii* f.a., sp. nov. and *Starmerella vitae* f.a., sp. no, isolated from flowers and bees, and transfer of related *Candida* species to the genus *Starmerella* as new combinations. Int J Syst Evol Microbiol..

[60] Sipiczki M (2015). *Starmerella syriaca* f.a., sp. nov., an osmotolerant yeast species isolated from flowers in Syria. Antonie Van Leeuwenhoek..

[61] Taniwaki MH, Pitt JI, Iamanaka BT, Massi FP, Fungaro MH, Frisvad JC (2015). *Penicillium excelsum* sp. nov. from the Brazil nut tree ecosystem in the Amazon Basin’. PLoS ONE..

[62] Thanh VN, Hien DD, Thom TT (2013). *Moniliella byzovii* sp. nov., a chlamydospore-forming black yeast isolated from flowers. Int J Syst Evol Microbiol..

[63] Chappell CR, Fukami T (2018). Nectar yeasts: a natural microcosm for ecology. Yeast.

[64] Letten AD, Dhami MK, Ke PJ, Fukami T (2018). Species coexistence through simultaneous fluctuation-dependent mechanisms. Proc Natl Acad Sci USA.

[65] Vannette RL, Fukami T (2014). Historical contingency in species interactions: towards niche-based predictions. Ecol Lett.

[66] Rering CC, Beck JJ, Hall GW, McCartney MM, Vannette RL (2018). Nectar-inhabiting microorganisms influence nectar volatile composition and attractiveness to a generalist pollinator. New Phytol.

[67] Rusell AL, Ashman T-L (2019). Associative learning of flowers by generalist bumble bees can be mediated by microbes on the petals. Behav Ecol.

[68] Schaeffer RN, Rering CC, Maalouf I, Beck JJ, Vannette RL (2019). Microbial metabolites elicit distinct olfactory and gustatory preferences in bumblebees. Biol Lett.

[69] Sobhy IS, Baets D, Goelen T, Herrera-Malaver B, Bosmans L, Van den Ende W, Verstrepen KJ, Wäckers F, Jacquemyn H, Lievens B (2018). Sweet scents: nectar specialist yeasts enhance nectar attraction of a generalist aphid parasitoid without affecting survival. Front Plant Sci..

[70] Sobhy IS, Goelen T, Herrera-Malaver B, Verstrepen KJ, Wäckers F, Jacquemyn H, Lievens B (2019). Associative learning and memory retention of nectar yeast volatiles in a generalist parasitoid. Anim Behav.

[71] Herrera CM, Pozo MI, Medrano M (2013). Yeasts in nectar of an early-blooming herb: sought by bumble bees, detrimental to plant fecundity. Ecology.

[72] Schaeffer RN, Irwin RE (2014). Yeasts in nectar enhance male fitness in a montane perennial herb. Ecology.

[73] Schaeffer RN, Mei YZ, Andicoechea J, Manson JS, Irwin RE (2017). Consequences of a nectar yeast for pollinator preference and performance. Funct Ecol.

[74] Good AP, Gauthier MP, Vannette RL, Fukami T (2014). Honey bees avoid nectar colonized by three bacterial species, but not by a yeast species, isolated from the bee gut. PLoS ONE.

[75] Bruce TJA, Midega CAO, Birkett MA, Pickett JA, Khan ZR (2010). Is quality more important than quantity? Insect behavioural responses to changes in a volatile blend after stemborer oviposition on an African grass. Biol Lett.

[76] Eisikowitch D, Lachance M-A, Kevan PG, Willis S, Collins-Thompson DL (1990). The effect of the natural assemblage of microorganisms and selected strains of the yeast *Metschnikowia reukaufii* in controlling the germination of pollen of the common milkweed *Asclepias syriaca*. Can J Bot.

[77] Vannette RL, Gauthier MP, Fukami T (2013). Nectar bacteria, but not yeast, weaken a plant-pollinator mutualism. Proc Biol Sci..

[78] Lenaerts M, Goelen T, Paulussen C, Herrera-Malaver B, Steensels J, Van den Ende W, Verstrepen KJ, Wäckers F, Jacquemyn H, Lievens B (2017). Nectar bacteria affect life history of a generalist aphid parasitoid by altering nectar chemistry. Funct Ecol.

[79] Tsuji K, Fukami T (2018). Community-wide consequences of sexual dimorphism: evidence from nectar microbes in dioecious plants. Ecology.

[80] Vannette RL, Fukami T (2016). Nectar microbes can reduce secondary metabolites in nectar and alter effects on nectar consumption by pollinators. Ecology.

[81] Tucker CM, Fukami T (2014). Environmental variability counteracts priority effects to facilitate species coexistence: evidence from nectar microbes. Proc Biol Sci..

