# Supplementary material for: Towards a better understanding of the role of nectar-inhabiting yeasts in plant–animal interactions
Source: Fungal Biol Biotechnol. 2020 Jan 8;7:1. doi: 10.1186/s40694-019-0091-8 (PMC6947986; doi:10.1186/s40694-019-0091-8)
Supplement: Supplementary file 1 — Additional file 1. Poster providing an overview of flower-inhabiting fungi and their effects on the host plant and flower visitors. [file 40694_2019_91_MOESM1_ESM.pdf]

Primers on Fungal Biology and Biotechnology

Flower mycology: towards a better understanding of the role of fungi in plant-animal interactions

FUNGAL BIOLOGY AND BIOTECHNOLOGY

Joon Klaps, Bart Lievens and Sergio Álvarez-Pérez

Flower-inhabiting fungi

- Flowers are initially sterile but rapidly colonized after anthesis by microorganisms from various sources, including the air, rain drops, dew, pollen from other flowers, and flower-visiting animals [1,2].
- Flower microbial communities are species poor relative to other plant microbiomes (e.g. the rhizosphere or the phylloplane), and they are often dominated by select yeasts and bacteria [1-5].
- The main fungal group inhabiting flowers are yeasts from the genus *Metschnikowia* [1-4]. Other flower-inhabiting yeasts and yeast-like fungi include species of the genera *Aureobasidium*, *Candida*, *Cryptococcus*, *Debaryomyces*, *Hanseniaspora*, *Kodamaea*, *Papiliotrema*, *Rhodotorula*, *Starmerella*, *Sporobolomyces*, and *Wickerhamiella*, whereas mycelial fungi are only rarely found in flowers [1,3,4].
- The physical and chemical characteristics of floral microhabitats have a filtering effect on microbial diversity, particularly nectar [1-7]. Dispersal limitation and microbe–microbe interactions can also determine the species composition of the flower microbiome [2,8,9].
- Although individual flowers are ephemeral, the collection of flowers on a plant acts as a microbial metacommunity that lasts longer than individual flowers while the plant is blooming [10]. Outside of the flowering season, flower-visiting animals may act as reservoirs of flower-inhabiting microbes [11].

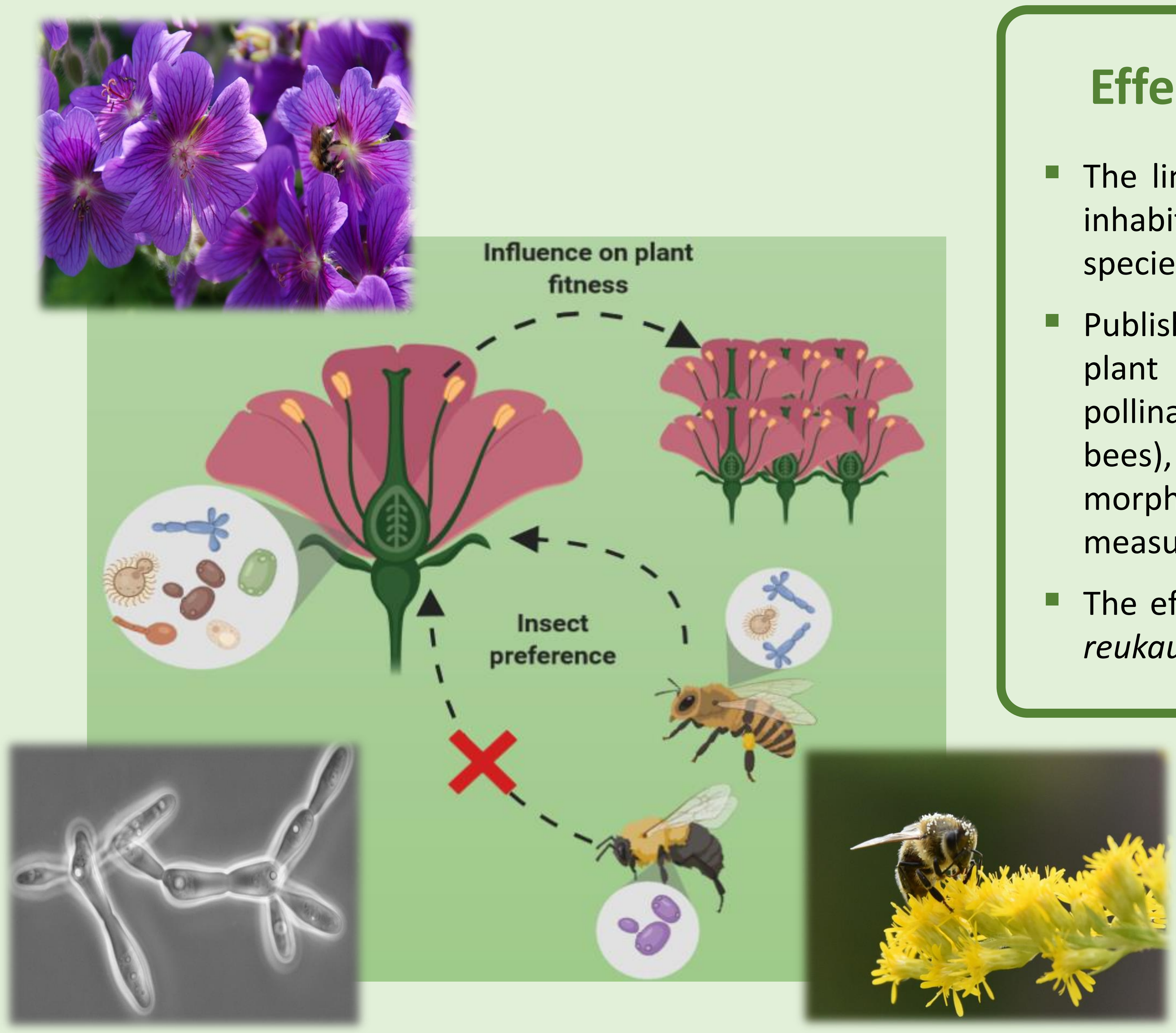

Overview of potential effects of floral yeasts.
(I) Influence on plant fitness, resulting in increased/decreased reproduction and offspring. (II) Alteration of pollinator's foraging behavior mediated by the production of VOCs.

Effects of floral fungi on their host plants

- The limited research carried out so far on the effects of flower-inhabiting microbes on plant fitness has focused on the yeast species *Metschnikowia reukaufii*.
- Published evidence suggests that the effect of *M. reukauffi* on plant fitness may depend not only on its direct effects on pollinators (e.g. increased attraction and longer visits of bumble bees), but also on plant specific attributes such as flower morphology, plant mating system, the component of reproduction measured, and the pollen limitation experienced [4,12,13].
- The effect(s) on plant fitness of flower inhabitants other than *M. reukauffi* remain(s) to be addressed in the future.

Effects of fungi on the animal visitors of flowers

- Flower-inhabiting microbes can produce species-specific blends of volatile organic compounds (VOCs) that alter the behavior of various pollinators and other floral visitors [14-18].
- The chemical cues produced by floral microbes can mediate both innate and learned components of insects preference. Learning of such cues is associative and reward context-dependent [16,17]. Furthermore, flower visitors can respond differentially to the olfactory *vs.* gustatory cues produced by nectar microbes [18].
- The modification of nectar's chemistry caused by flower-inhabiting microbes can affect the life history parameters (e.g. longevity and survival) of floral visitors, although the actual effects are species-dependent [15,16,19].

Sources and Further Reading

[1] Aleklett K, *et al.* (2014) *Botany*, 92(4):253–66.
[2] Álvarez-Pérez S, *et al.* (2019) *Trends Plant Sci*, 24(5):393–401.
[3] Klaps J (2019) Analysis of yeast diversity in floral microhabitats from *Metrosideros polymorpha* Gaud. Master Thesis, KU Leuven.
[4] Pozo M, *et al.* (2015) Impact of microorganisms on the nectar chemistry, pollinator attraction and plant fitness. *In:* Peck RL, editor. Nectar: Production, chemical composition and benefits to animals and plants. New York, NY, USA: Nova Science Publishers Inc.
[5] Junker RR & Keller A (2015) *FEMS Microbiol Ecol*, 91(9):fiv097.
[6] Herrera CM, *et al.* (2010) *Proc Biol Sci*, 277(1682):747–54.
[7] Lievens B, *et al.* (2015) *Environ Microbiol*, 17(2):278–98.
[8] Hausmann SL, *et al.* (2017) *FEMS Microbiol Ecol*, 93(12):fix150.
[9] Vannette RL & Fukami T (2017) *Ecol Lett*, 20(7):901–10.
[10] Toju H, *et al.* (2018) *Oikos*, 127(3):345–52.
[11] Pozo MI, *et al.* (2018) *FEMS Microbiol Ecol*, 94(12):fiy196.
[12] Herrera CM, *et al.* (2013) *Ecology*, 94(2):273–9.
[13] Schaefer RN & Irwin RE (2014) *Ecology*, 95(7):1792–8.
[14] Rering CC, et al. (2018) *New Phytol*, 220(3):750–59.
[15] Sobhy IS, *et al.* (2018) *Front Plant Sci*, 9:1009.
[16] Sobhy IS, *et al.* (2019) *Anim Behav*, 153:137–46.
[17] Russell AL & Ashman T-L (2019) *Behav Ecol*, 30(3):746–55.
[18] Schaeffer RN, *et al.* (2019) *Biol Lett*, 15(7):20190132.
[19] Lenaerts M, *et al.* (2017) *Funct Ecol*, 31(11):2061–69.
